# LncRNA HOXA10-AS as a novel biomarker and therapeutic target in human cancers

**DOI:** 10.3389/fmolb.2024.1520498

**Published:** 2025-01-03

**Authors:** Xin Hu, Yong Wang, Sijia Zhang, Xiaosi Gu, Xiaoyu Zhang, Lianlian Li

**Affiliations:** ^1^ Department of Immunology, School of Clinical and Basic Medical Sciences, Shandong First Medical University, Jinan, Shandong, China; ^2^ Shandong Provincial Engineering Research Center for Bacterial Oncolysis and Cell Treatment, Jinan, Shandong, China; ^3^ Laboratory of Metabolism and Gastrointestinal Tumor, The First Affiliated Hospital of Shandong First Medical University, Jinan, Shandong, China

**Keywords:** long non-coding RNA, HOXA10-AS, signaling pathway, biomarker, therapeutic target

## Abstract

Long non-coding RNAs (lncRNAs) are crucial regulatory molecules that participate in numerous cellular development processes, and they have gathered much interest recently. HOXA10 antisense RNA (HOXA10-AS, also known as HOXA-AS4) is a novel lncRNA that was identified to be dysregulated in some prevalent malignancies. In this review, the clinical significance of HOXA10-AS for the prognosis of various cancers is analyzed. In addition, the major advances in our understanding of the cellular biological functions and mechanisms of HOXA10-AS in different human cancers are summarized. These cancers include esophageal carcinoma (ESCA), gastric cancer (GC), glioma, laryngeal squamous cell carcinoma (LSCC), acute myeloid leukemia (AML), lung adenocarcinoma (LUAD), nasopharyngeal carcinoma (NPC), oral squamous cell carcinoma (OSCC), and pancreatic cancer. We also note that the aberrant expression of HOXA10-AS promotes malignant progression through various underlying mechanisms. In conclusion, HOXA10-AS is expected to serve as an ideal clinical biomarker and an effective cancer therapy target.

## 1 Introduction

Cancer has high incidence and fatality rate, and is steadily overtaking stroke and coronary heart disease as the primary cause of human mortality. There were approximately 9.7 million cancer deaths and 20.0 million new cases worldwide in 2022, according to the most recent global data (Bray et al., 2024). Escalating cancer incidence and fatality rates emphasize the need for innovative methods to detect, improve prognoses, and provide precision therapies.

Several non-coding RNAs have recently been shown to be involved in various tumors, and have been used as clinical biomarkers and therapeutic targets. These RNAs include long non-coding RNAs (lncRNAs). LncRNAs are RNA types that do not encode proteins and are longer than 200 nucleotides (nt) in length. They are primarily found in the nucleus and cytoplasm (Statello et al., 2021). LncRNAs affect transcription, mRNA turnover, and translation. These functions are primarily achieved through changes in their relative stability and interactions with nucleic acids or RNA-binding proteins (RBPs) in the cis or trans forms (Herman, Tsitsipatis, and Gorospe, 2022). Cancer initiation and progression have been linked to lncRNAs, and they can affect cancer cell differentiation, epithelial-mesenchymal transition (EMT), energy metabolism, oxidative stress, autophagy, chemoresistance, and immunity (McCabe and Rasmussen, 2021; Tan et al., 2021; Liu et al., 2022; Yao et al., 2022; Ahmad et al., 2023). Notably, lncRNAs have shown potential as diagnosis and prognosis indicators, as well as therapeutic cancer targets (Liu et al., 2024b; Ramnarine et al., 2019; Ao et al., 2023; Kuang et al., 2022; Li et al., 2021a).

**FIGURE 1:**
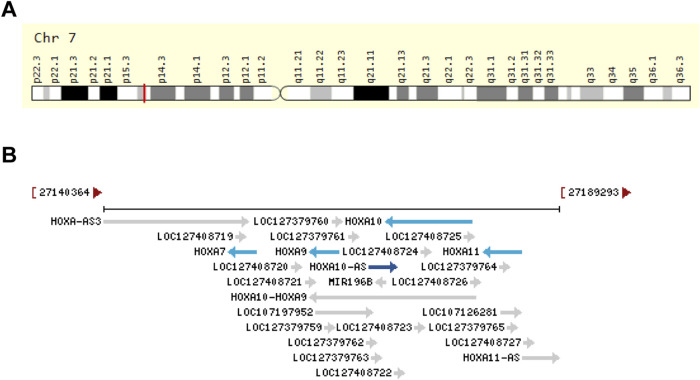
Genomic view of HOXA10-AS in terms of **(A)** its genomic location as extracted from GeneCards database (https://www.genecards.org/cgi-bin/carddisp.pl?gene=HOXA10-AS&keywords=HOXA10-AS) (Stelzer et al., 2016), and **(B)** its genomic context as extracted from the NCBI database (https://www.ncbi.nlm.nih.gov/gene/100874323).

Among these lncRNAs, HOXA10 antisense RNA (HOXA10-AS, also known as HOXA-AS4) has garnered attention as a novel antisense lncRNA due to its aberrant expression observed in human malignancies (Al-Kershi et al., 2019; Chen et al., 2022; Kuai et al., 2023; Li et al., 2022; Sheng et al., 2018; Wang, 2021; Wang, Nie, and Zhu, 2022; Wu et al., 2022; Yan et al., 2020; Zhao et al., 2024). HOXA10-AS is transcribed from the reverse strand of human chromosome 7q15.2 (Figure 1A) at coordinates 27168899 to 27171915, spans three exons, and has a length of 3,017 nt (source: https://www.ncbi.nlm.nih.gov/gene/100874323). HOXA10-AS originates from the antisense strand of HOXA10 and shares this genomic location with miR-196b and HOXA10 (Figure 1B). HOXA10 is highly expressed in gastric cancer (GC) and has increased cell proliferation and EMT (Song and Zhou, 2021). A low expression of miR-196b has been shown to effect human choriocarcinoma cell proliferation, migration, and invasion (Guo et al., 2019). This association indicates that HOXA10-AS could play a potential role in tumor onset and progression. An increasing amount of research has also shown that HOXA10-AS is highly expressed in most tumor cells and tissues. Further research has demonstrated that HOXA10-AS also plays a regulatory role in signaling pathways (Sheng et al., 2018; Wang, 2021; Wang, Nie, and Zhu, 2022; Wu et al., 2022; Zhao et al., 2024). HOXA10-AS has the potential to function as a biomarker for diagnosis and prognosis, and could be a therapeutic cancer target.

In this review, we systematically searched PubMed, the Web of Science, and Google Scholar for articles related to HOXA10-AS to summarize the biological functions and molecular mechanisms of HOXA10-AS in cancer. The online databases were also used to analyze the HOXA10-AS differential expression in pan-cancer and the diagnostic and prognostic value of HOXA10-AS in different human cancers.

## 2 HOXA10-AS gene expressions in human cancers

Numerous studies have reported on the aberrant expression of the lncRNA HOXA10-AS in many human cancers. These cancers include GC (Li et al., 2022), pancreatic cancer (Wu et al., 2022), esophageal carcinoma (ESCA) (Kuai et al., 2023), oral squamous cell carcinoma (OSCC) (Chen et al., 2022; Wang, 2021; Yan et al., 2020), acute myeloid leukemia (AML) (Al-Kershi et al., 2019), lung adenocarcinoma (LUAD) (Sheng et al., 2018), nasopharyngeal carcinoma (NPC) (Wang, Nie, and Zhu, 2022), laryngeal squamous cell carcinoma (LSCC) (Zhao et al., 2024), and gliomas (Dong et al., 2018; Isaev et al., 2021; Li et al., 2021b) (Table 1).

**TABLE 1:** Expression of HOXA10-AS and its relationship with clinical characteristics and survival in human cancers^a^.

Tumor type	Expression	Model			Clinical Characteristics	Survival indicator	Biomarker	Ref.
		Tissue	Cell line	Main subcellular location				
Esophageal carcinoma	Upregulated	UALCAN	TE-1, Eca-109, Kyse-150	Cytoplasm (Eca-109)	—	—	—	Kuai et al. (2023)
Gastric cancer	Upregulated	Human tissues, ENCORI	SNU-1, AGS, HGC-27	—	—	OS	Prognostic	Li et al. (2022)
Glioma	Upregulated	Human tissues	A172, U251	—	Malignancy grade	—	Prognostic	Dong et al. (2018)
		TCGA	G797, G432	—	Malignancy grade	OS		Isaev et al. (2021)
		TCGA	—	—	—	OS		Li et al. (2021b)
KMT2A-rearrangede acute myeloid leukemia	Upregulated	Human tissues, TCGA	EOL-1, MOLM-13, MV4-11, NOMO, THP-1, SHI-1, ML-2	Cytoplasm (EOL, MOLM-13, MV4-11, NB-4)	—	OS	Prognostic	Al-Kershi et al. (2019)
Lung adenocarcinoma	Upregulated	Human tissues, TCGA	A549, H1299, H1975, PC9, SPC-A1	—	—	OS	Prognostic	Sheng et al. (2018)
Laryngeal squamous cell carcinoma	Upregulated	Human tissues, TCGA	AMC-HN-8, TU686, TU212	—	—	OS	Prognostic	Zhao et al. (2024)
Nasopharyngeal carcinoma	Upregulated	GEPIA	C666-1, 5-8F, SUNE-1	Cytoplasm (C666-1, SUNE-1)	—	—	—	Wang, Nie, and Zhu (2022)
Oral squamous cell carcinoma	Upregulated	Human tissues	Tca8113	—	TNM stage	OS	Prognostic	Wang (2021)
		Human tissues, TCGA	SAS, SCC25	—	—	OS	Prognostic	Chen et al. (2022)
		TCGA	CAL27, SCC9	—	—	OS	Prognostic	Yan et al. (2020)
Pancreatic cancer	Upregulated	TCGA	BxPC-3, SW 1990, PANC-1, CFPAC-1, AsPC-1, T3M4	—	—	OS	Prognostic	Wu et al. (2022)

^a^ OS, overall survival; TNM, stage, tumor node metastasis stage; Ref, reference.

**FIGURE 2:**
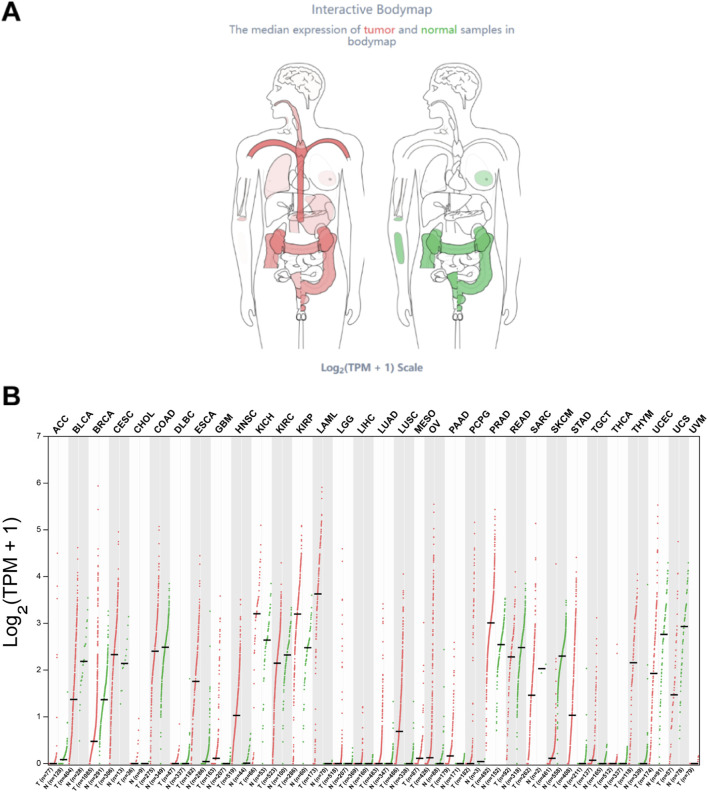
Expression profile of HOXA10-AS across all tumor and normal human tissues as obtained using the GEPIA two database. The median expression of HOXA10-AS in tumor and normal samples is shown in an interactive bodymap **(A)**. A thorough profile of gene expression is presented in the form of dot plot **(B)**, and each dots represent expression of samples.

The HOXA10-AS expression in pan-cancers was then more thoroughly explored by analyzing the interactive bodymap and the HOXA10-AS RNA transcript expression levels in pan-cancers using GEPIA2 (http://gepia2.cancer-pku.cn/) (Tang et al., 2019) (Figure 2A−B). The interactive bodymap indicated that HOXA10-AS was more highly expressed in cancers of the head and neck, lung, stomach, pancreas, kidney, cervix uteri, prostate, and blood system as compared to normal tissue expressions (Figure 2A). In the tumor tissues, the highest RNA transcript expression was found in acute myeloid leukemia (LAML), while in the paired normal tissues, the highest RNA transcript expression was found in uterine carcinosarcoma (UCS) (Figure 2B).

**FIGURE 3:**
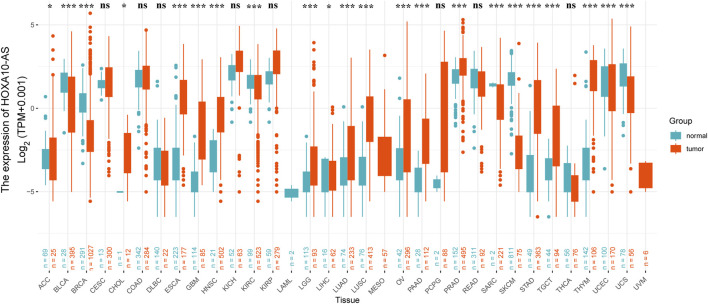
Relative expression level of HOXA10-AS in different cancers based on the UCSC Xena datasets. Not significant (ns), *p < 0.05, **p < 0.01, ***p < 0.001.

The HOXA10-AS expression between tumor and normal tissues was then comprehensively compared, and we also assessed the HOXA10-AS expression profile in 33 cancer types using the University of Santa Cruz (UCSC) Xena website (https://xenabrowser.net/datapages/) (Goldman et al., 2020) (Figure 3). HOXA10-AS expression was significantly different across various cancers, including cholangiocarcinoma (CHOL), ESCA, glioblastoma multiforme (GBM), HNSC, brain lower grade glioma (LGG), liver hepatocellular carcinoma (LIHC), LUAD, lung squamous cell carcinoma (LUSC), ovarian serous cystadenocarcinoma (OV), pancreatic adenocarcinoma (PAAD), prostate adenocarcinoma (PRAD), STAD, testicular germ cell tumors (TGCT), THYM, adrenocortical carcinoma (ACC), bladder urothelial carcinoma (BLCA), breast invasive carcinoma (BRCA), kidney renal clear cell carcinoma (KIRC), sarcoma (SARC), SKCM, uterine corpus endometrial carcinoma (UCEC), and UCS. Due to these varied expression patterns across different cancer types, the HOXA10-AS expression levels may be clinically relevant for predicting disease onset and progression.

## 3 The HOXA10-AS clinical significance in cancers

HOXA10-AS has the potential to serve as a novel predictive biomarker, and its expression has been associated with several clinical features of many cancers (Table 1). The HOXA10-AS expression level is related to clinicopathological features. For instance, the HOXA10-AS expression in tumor tissues was shown to be positively correlated with tumor node metastasis stages (Wang, 2021) and the malignancy status (Dong et al., 2018; Isaev et al., 2021). In addition, the abnormal expression of HOXA10-AS is linked to many cancer prognoses. Higher HOXA10-AS expressions have been linked to shorter survival times in GC (Li et al., 2022), pancreatic cancer (Wu et al., 2022), leukemia (Al-Kershi et al., 2019), OSCC (Wang, 2021; Yan et al., 2020), LUAD (Sheng et al., 2018), LSCC (Zhao et al., 2024), and glioma (Isaev et al., 2021).

**FIGURE 4:**
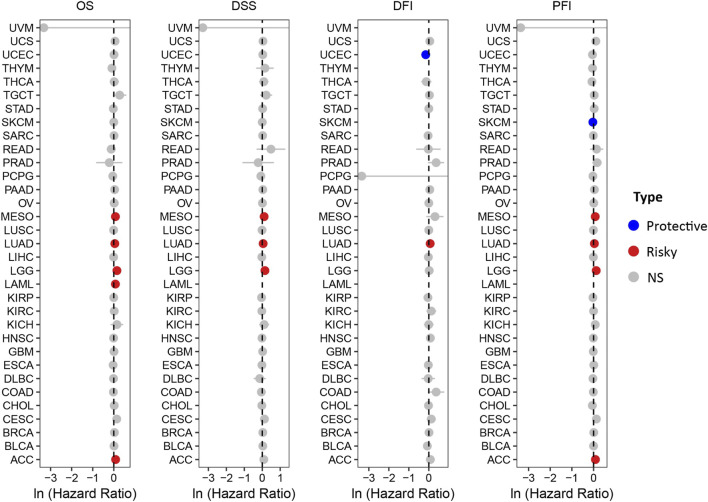
Forest plots showing the relationship between HOXA10-AS and OS/DSS/DFI/PFI in pan-cancer using the UCSC Xena datasets.

The prognostic significance of HOXA10-AS in various other cancers was then further explored. The UCSC Xena datasets were extensively evaluated to elucidate the relationship between HOXA10-AS expression and prognostic indicators that included the overall survival (OS), the disease-specific survival (DSS), the disease-free interval (DFI), and the progression free interval (PFI) (Figure 4). Upregulated HOXA10-AS levels indicated worse OS, DSS, and PFI in mesothelioma and LGG, shorter OS, DSS, DFI, and PFI in LUAD, worse OS in LAML and shorter OS and PFI in ACC, and better DFI in UCEC and PFI in SKCM.

**FIGURE 5:**
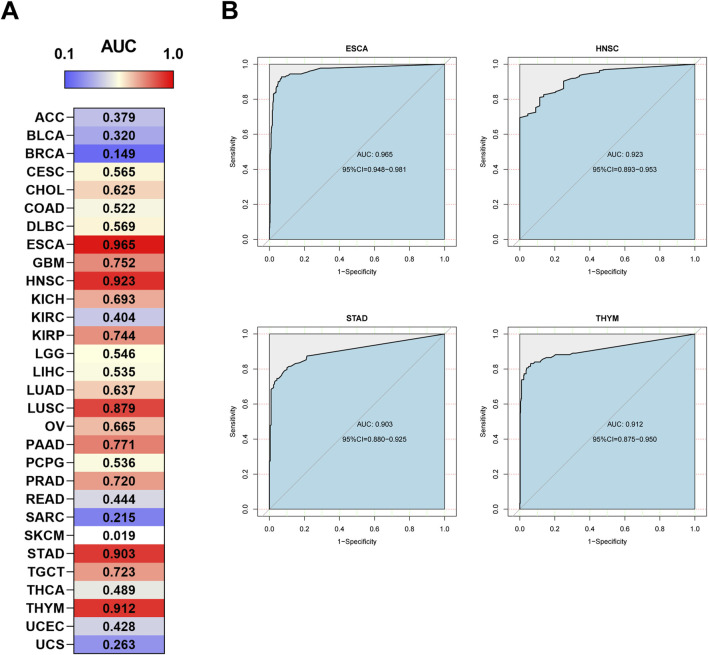
Diagnostic ROC curves of **(A)** HOXA10-AS expression for differentiating cancer from normal tissues, and **(B)** HOXA10-AS expression in ESCA, HNSC, STAD and THYM cancer types, which have a stronger diagnostic value when using the UCSC Xena datasets.

A receiver operating characteristic (ROC) curve analysis was also performed. The results showed that HOXA10-AS has potential diagnostic value in some cancer types (Figure 5A), particularly in ESCA, HNSC, STAD, and THYM, in which the area under the curve (AUC) exceeded 0.9 (Figure 5B). These results demonstrated that HOXA10-AS can be used as a potential diagnostic and prognostic biomarker in a wide range of tumors.

## 4 HOXA10-AS biological functions and regulatory mechanisms in various tumors

**FIGURE 6:**
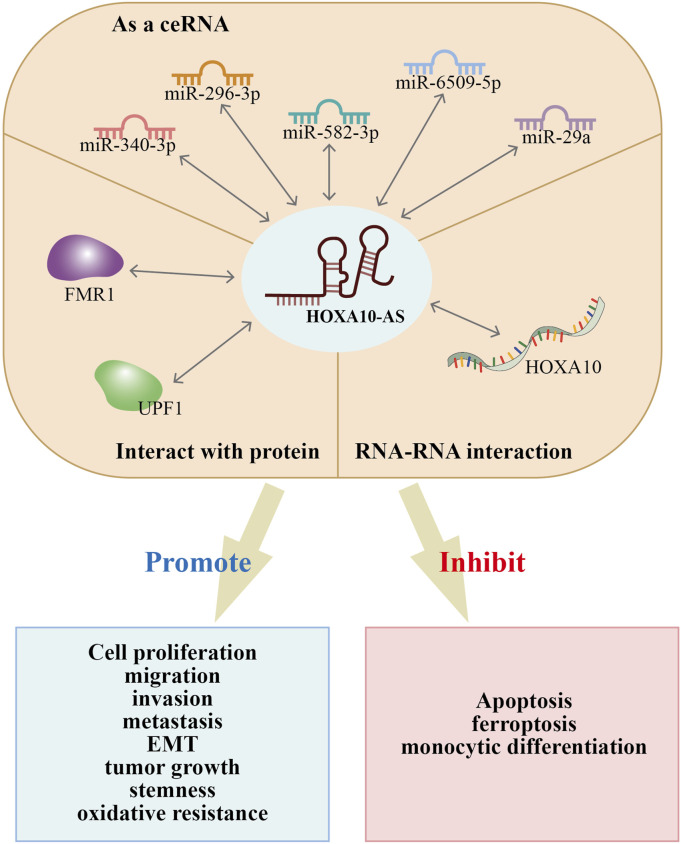
Primary mechanisms of HOXA10-AS in the occurrence and progression of multiple tumors.

**FIGURE 7:**
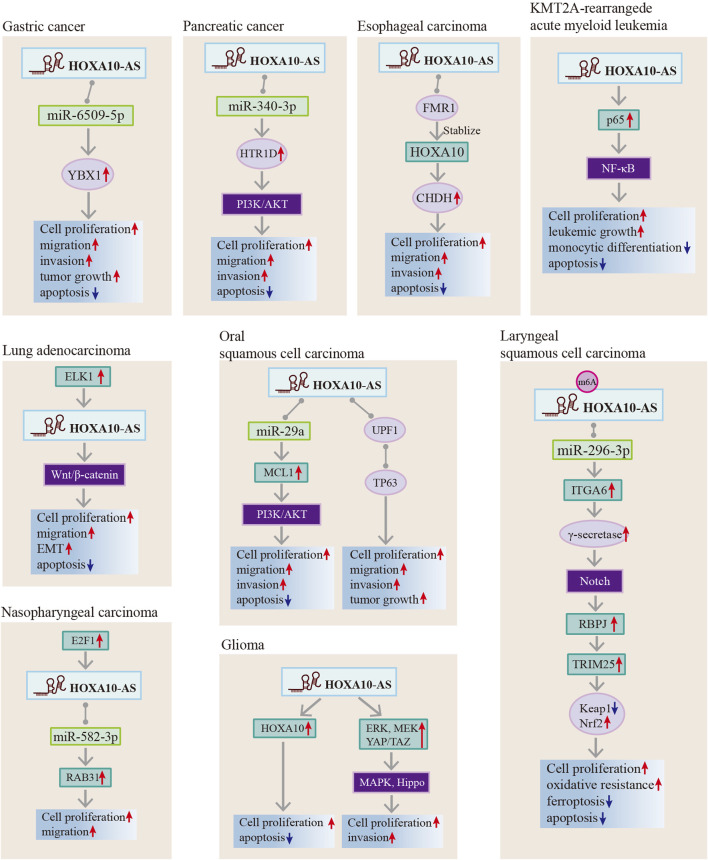
Regulatory mechanisms of HOXA10-AS in gastric cancer, pancreatic cancer, esophageal carcinoma, oral squamous cell carcinoma, KMT2A-rearrangede acute myeloid leukemia, lung adenocarcinoma, nasopharyngeal carcinoma, laryngeal squamous cell carcinoma, and gliomas.

HOXA10-AS has been reported to involved in the regulation of biological functions that include cell proliferation, apoptosis, ferroptosis, monocytic differentiation, migration, invasion, metastasis, EMT, stemness, tumor growth, and oxidative resistance. All of these factors impact cancer onset and progression (Table 2; Figure 6). Recent studies have investigated the functional roles of HOXA10-AS in many cancer types such as ESCA, GC, glioma, AML, LUAD, LSCC, NPC, OSCC, and pancreatic cancer. The HOXA10-AS regulatory mechanisms in these cancers are shown in Figure 7.

**TABLE 2:** The biological function and mechanisms of lncRNA HOXA10-AS in multiple human cancers^a^.

Cancer type	Role	Experiment	Function	Action Mechanism of HOXA10-AS	Related Molecule/Signal	Ref.
Esophageal carcinoma	Oncogenic	*In vitro*	Promotes cells proliferation, migration, and invasion; inhibits cell apoptosis	Interaction with protein	FMR1, HOXA10, CHDH	Kuai et al. (2023)
Gastric cancer	Oncogenic	*In vitro* and *in vivo*	Promotes cell proliferation, migration, invasion, and tumor growth; inhibits cell apoptosis	As a ceRNA	miR-6509-5p, YBX1	Li et al. (2022)
Glioma	Oncogenic	*In vitro*	Promotes cell proliferation; inhibits cell apoptosis	RNA–RNA interaction	HOXA10	Dong et al. (2018)
		*In vitro* and *in vivo*	Promotes cell proliferation and invasion	—	ERK, MEK, YAP/TAZ; MAPK pathway; Hippo pathway	Isaev et al. (2021)
KMT2A-rearrangede acute myeloid leukemia	Oncogenic	*In vitro* and *in vivo*	Promotes cell proliferation and leukemic growth; inhibits cell apoptosis and monocytic differentiation	—	P65; NF-κB pathway	Al-Kershi et al. (2019)
Lung adenocarcinoma	Oncogenic	*In vitro*	Promotes cell proliferation, migration, and EMT; inhibits cell apoptosis	—	ELK1; Wnt/β-catenin pathway	Sheng et al. (2018)
Laryngeal squamous cell carcinoma	Oncogenic	*In vitro* and *in vivo*	Promotes cell proliferation and activates oxidative resistance of cancer cells; inhibits cell apoptosis and ferroptosis	As a ceRNA	miR-296-3p, ITGA6, TRIM25, RBPJ, Keap1, Nrf2; Notch pathway	Zhao et al. (2024)
Nasopharyngeal carcinoma	Oncogenic	*In vitro*	Promotes cell proliferation and migration	As a ceRNA	E2F1, miR-582-3p, RAB31	Wang, Nie, and Zhu (2022)
Oral squamous cell carcinoma	Oncogenic	*In vitro* and *in vivo*	Promotes cell proliferation, migration, invasion, stemness, tumor growth and metastasis	As a ceRNA	miR-29a, MCL-1; PI3K/AKT pathway	Wang (2021)
		*In vitro* and *in vivo*	Promotes cell proliferation, migration, invasion, and tumor growth	Interaction with protein	UPF1, TP63	Chen et al. (2022)
		*In vitro*	Promotes cell proliferation	—	—	Yan et al. (2020)
Pancreatic cancer	Oncogenic	*In vitro*	Promotes cells proliferation, migration, and invasion; inhibits cell apoptosis	As a ceRNA	miR-340-3p, HTR1D; PI3K/AKT pathway	Wu et al. (2022)

^a^ EMT, epithelial-mesenchymal transition; Ref, reference.

### 4.1 Esophageal carcinoma

ESCA accounted for 4.6% of all cancer-related deaths in 2022, with the highest incidence occurring in East Asia (Bray et al., 2024). Molecular targeted therapy research is still ongoing due to its poor prognosis (Deboever et al., 2024). ESCA cell lines have high expressions of HOXA10-AS (Kuai et al., 2023). HOXA10-AS has been demonstrated to increase cell proliferation, migration, and invasion while reducing cell apoptosis in ESCA cells and to facilitate ESCA tumor growth in tumor xenograft models (Kuai et al., 2023). Mechanistically, HOXA10-AS interacts with the RNA-binding protein FMR1, diminishing FMR1’s ability to stabilize HOXA10 by reducing its binding affinity to FMR1 HOXA10. This reduction in binding subsequently enhances enhancing CHDH expression (Kuai et al., 2023). This study suggests that HOXA10-AS could be a therapeutic target for ESCA treatment.

### 4.2 Gastric cancer

GC remains prevalent and is the leading cause of global cancer deaths. The complex molecular characteristics of GC require additional investigation. The current clinical targeted drugs for GC lack single agent frontline efficacy. Therefore, an exploration of non-coding RNAs may provide new targeted choices and improve individual treatments (Zeng and Jin, 2022; Guan et al., 2023). HOXA10-AS is upregulated in GC tissues, and its high expression is correlated with a poor prognosis (Li et al., 2022). Elevated HOXA10-AS mRNA expression levels have been observed in human GC cell lines, including Sun-1, AGS, and HGC-27 (Li et al., 2022). Functional studies have demonstrated that HOXA10-AS affects GC cell proliferation, apoptosis, migration and invasion *in vitro*, and it controls GC tumorigenesis in mouse xenograft models (Li et al., 2022). Mechanistically, HOXA10-AS promotes GC development by binding to miR-6509-5p, thereby indirectly regulating YBX-1 (Li et al., 2022). Thus, HOXD10 can serve as a novel biomarker for the detection of GC or as an attractive GC therapeutic target.

### 4.3 Gliomas

Glioma is the most common malignant primary brain tumor, and it has a high mortality rate. Tumor growth is controlled by targeting various molecules in the signaling pathways combined with immunotherapy (Yang et al., 2022). Increased HOXA10-AS levels have been observed in glioma tissues and cell lines, as it has a positive association with higher glioma grades (Dong et al., 2018; Isaev et al., 2021). It is noteworthy that HOXA10-AS serves as a prognostic glioma biomarker, and its high expression is associated with isocitrate dehydrogenase (IDH) mutations and neurodevelopmental pathway dysregulation (Isaev et al., 2021; Li et al., 2021b). *In vitro* experiments have shown that HOXA10-AS leads to the promotion of cell proliferation (Dong et al., 2018; Isaev et al., 2021), invasion (Isaev et al., 2021), and apoptosis inhibition (Dong et al., 2018). In addition, *in vivo* investigations have validated that HOXA10-AS overexpression increases the tumorigenic potential of glioma cells (Isaev et al., 2021). Mechanistically, HOXA10-AS exerts oncogenic effects in gliomas by targeting HOXA10 and repressing HOXA10 expression (Dong et al., 2018). Additionally, HOXA10-AS promotes glioma cell proliferation and the loss of contact inhibition through MAPK signaling pathway activation and Hippo signaling pathway inhibition (Isaev et al., 2021). Collectively, these studies indicate that HOXA10-AS has pro-metastatic effects in glioma and could be a promising glioma prognostic biomarker.

### 4.4 KMT2A-rearrangede acute myeloid leukemia

KMT2A-rearrangede acute myeloid leukemia (KMT2A-r AML) is an acute leukemia characterized by a chromosomal translocation of the KMT2A gene, and it has poor outcomes (Bourgeois et al., 2024). KMT2A-r AML also shows upregulation of HOXA cluster genes (Bourgeois et al., 2024), and HOXA10 is a target of KMT2A-fusions (Di Mambro et al., 2023). The HOXA10-AS expression level was found to be high in hematopoietic stem cells and KMT2A-r cell lines (Al-Kershi et al., 2019). In addition, HOXA10-AS increases the prognostic value of AML as a non-independent prognostic marker (Al-Kershi et al., 2019). HOXA10-AS knockdown inhibited 3 KMT2A-r cell line proliferation (EOL-1, MOLM-13, and MV4-11) and induced EOL cell line apoptosis (Al-Kershi et al., 2019). In contrast, HOXA10-AS upregulation increased leukemic growth and blocked monocytic differentiation (Al-Kershi et al., 2019). The HOXA10-AS oncogenic roles were achieved by elevating p65 phosphorylation, thus activating the NF-κB pathway (Al-Kershi et al., 2019). Therefore, these results suggest that the aberration of HOXA10-AS has pro-metastatic effects in KMT2A-r leukemias and that HOXA10-AS inhibition may be a viable strategy for KMT2A-r leukemias.

### 4.5 Lung adenocarcinoma

The most typical type of lung cancer is non-small cell lung cancer, LUAD being its most frequently diagnosed subtype (Thai et al., 2021; Haga et al., 2023). HOXA10-AS is highly expressed in LUAD cell lines and tissues, and is associated with a worse prognosis in LUAD patients (Sheng et al., 2018). Functional studies have demonstrated that HOXA10-AS knockdown affects LUAD cell proliferation, apoptosis, and migration (Sheng et al., 2018). HOXA10-AS upregulation promotes proliferation and migration and inhibits apoptosis by activating the Wnt/β-catenin signaling pathway. Upregulation also induces epithelial-mesenchymal transition by decreasing E-cadherin expression and increasing N-cadherin expression (Sheng et al., 2018). This suggests that HOXA10-AS may have a role in lung cancer as an oncogenic gene and could be a novel therapeutic LUAD target.

### 4.6 Laryngeal squamous cell carcinoma

LSCC is a common head and neck cancer that requires biomarkers for early diagnosis and surveillance of disease recurrence and metastasis (Sun et al., 2024). The HOXA10-AS expression is considerably upregulated in both LSCC specimens and LSCC cell lines, and its upregulation is positively associated with poor OS in LSCC patients (Zhao et al., 2024). *In vitro* and *in vivo* studies have revealed that increased HOXA10-AS promotes cell proliferation, activates cancer cell oxidative resistance, and inhibits LSCC cell apoptosis and ferroptosis (Zhao et al., 2024). Mechanistically, HOXA10-AS subjected to m6A modification upregulates ITGA6 expression through the sponging of miR-296-3p (Zhao et al., 2024). In addition, elevated ITGA6 activates γ-secretase activity in the Notch pathway and thus upregulates TRIM25 by upregulating the RBPJ transcription factor (Zhao et al., 2024). Increased TRIM25 enhances anti-oxidative stress in LSCC cells by degrading Keap1 and activating Nrf2 (Zhao et al., 2024). These studies suggest that HOXA10-AS can serve as a LSCC prognostic marker and may play a role in the anti-oxidative stress process in LSCC cells.

### 4.7 Nasopharyngeal carcinoma

NPC is a malignant tumor of the head and neck with unique epidemiological features. It is primarily associated with genetic susceptibility, Epstein-Barr virus infection, and environmental and dietary factors (Liu et al., 2024a). Elevated HOXA10-AS expressions have been observed in both NPC tissues and NPC cell lines (Wang, Nie, and Zhu, 2022). E2F1 binds to the promoter of HOXA10-AS and induces HOXA10-AS dysregulation (Wang, Nie, and Zhu, 2022). HOXA10-AS serves as a sponge for miR-582-3p to upregulate RAB31 expression, thereby promoting NPC cell proliferation and migration (Wang, Nie, and Zhu, 2022). These findings suggest that HOXA10-AS confers various oncogenic properties on NPC cells and its upstream and downstream molecules could be viable NPC treatment methods.

### 4.8 Oral squamous cell carcinoma

OSCC is a common head and neck cancer subtype, and the prospects for long-term patient survival remain dismal (Johnson et al., 2020). Only the discovery of new predictive biomarkers will provide new approaches to OSCC treatment. HOXA10-AS is increased in OSCC tissues compared to adjacent normal oral tissues (Wang, 2021) and is overexpressed in OSCC cell lines (CAL27, SCC9, Tca8113, SAS, SCC25) (Yan et al., 2020; Wang, 2021; Chen et al., 2022). In addition, HOXA10-AS upregulation has been found to be positively correlated with a worse prognosis in OSCC patients and a higher histological tumor grade (Yan et al., 2020; Wang, 2021; Chen et al., 2022). *In vivo* and *in vitro* studies have shown that HOXA10-AS induces OSCC malignant behavior by the promotion of OSCC cell proliferation, migration, invasion, stemness, tumor growth, and metastasis (Yan et al., 2020; Wang, 2021; Chen et al., 2022). Additionally, a high expression of HOXA10-AS affects chemotherapeutic drug sensitivity (Wang, 2021). Mechanistically, it was found that HOXA10-AS as a ceRNA upregulates MCL-1 by sponging miR-29a. This results in the activation of the PI3K/AKT signaling pathway (Wang, 2021). Furthermore, HOXA10-AS promotes tumor growth by providing a scaffold for the regulation of TP63 in a post-transcriptional manner (Chen et al., 2022). These results imply that HOXA10-AS is an essential regulator of OSCC progression and can be considered an indicator in differentiating the different OSCC clinical tumor grades.

### 4.9 Pancreatic cancer

Pancreatic cancer is an insidious and highly malignant cancer that threatens human life. Early screening is crucial and there is an urgent need to find new biomarkers for surveillance (Stoffel, Brand, and Goggins, 2023). Some lncRNAs have been found to be pro-oncogenic in pancreatic cancer and can be used as new biomarkers (da Paixão et al., 2022). HOXA10-AS is dramatically increased in pancreatic cancer tissues as well as in pancreatic adenocarcinoma cell lines (Wu et al., 2022). Functionally, the aberrant expression of HOXA10-AS promotes PDAC cell proliferation, migration, and invasion and inhibits cell apoptosis (Wu et al., 2022). Mechanistically, HOXA10-AS knockdown effectively impedes HTR1D-induced malignant progression in PANC-1 and CFPAC-1 pancreatic cancer cells by acting as ceRNA for sponging miR-340-3p (Wu et al., 2022). The results imply that HOXA10-AS is an essential regulator of pancreatic cancer cell progression and could be used as a therapeutic pancreatic cancer target.

## 5 Conclusion and discussion

LncRNAs have received widespread attention in recent years, and the number of identified annotated lncRNAs now exceeds 20,000 (Frankish et al., 2023). LncRNAs have been found to be involved in cancer development as oncogenes or tumor-suppressor genes (Bhan, Soleimani, and Mandal, 2017). An in-depth understanding of the molecular mechanism of lncRNAs in cancers is important for clinical treatment.

HOXA10-AS is an emerging lncRNA that has recently attracted much interest in the carcinogenesis field. LncRNA HOXA10-AS exhibits dysregulated expression in pan-cancers (Figure 3) and has been shown to be upregulated in solid and hematological tumors (Table 1). In addition, HOXA10-AS expression levels have been shown to be associated with several cancer clinical features such as malignancy grades, tumor node metastasis stages, and the OS (Table 1). Higher HOXA10-AS expression indicates more advanced cancer degrees in OSCC (Wang, 2021) and glioma (Dong et al., 2018; Isaev et al., 2021). Moreover, HOXA10-AS is frequently implicated in several biological cancer processes. HOXA10-AS overexpression promotes cell proliferation, migration, invasion, stemness, metastasis, EMT, oxidative resistance, and tumor growth. Furthermore, HOXA10-AS overexpression also inhibits cell apoptosis, ferroptosis, and monocytic differentiation (Al-Kershi et al., 2019; Chen et al., 2022; Dong et al., 2018; Isaev et al., 2021; Kuai et al., 2023; Li et al., 2022; Li et al., 2021b; Sheng et al., 2018; Wang, 2021; Wang, Nie, and Zhu, 2022; Wu et al., 2022; Yan et al., 2020; Zhao et al., 2024). These findings imply that HOXA10-AS is an essential participant in cancer onset and development due to its differential expression and biological functions.

A thorough analysis of the data from the UCSC Xena datasets, showed that HOXA10-AS could be utilized as both a diagnostic and prognostic biomarker for a variety of malignancies. HOXA10-AS has a strong diagnostic potential for differentiating between cancer and normal tissues, especially in ESCA, HNSC, STAD, and THYM. In terms of prognosis, HOXA10-AS showed high expressions in MESO, LUAD, LGG, LAML, and ACC indicated a poor prognosis, except for in UCEC and SKCM, where it is linked to a good prognosis. These results indicated that HOXA10-AS possesses a good diagnostic and prognostic value in cancers and is expected to play a crucial role in personalized diagnosis and treatments.

HOXA10-AS possesses different regulatory mechanisms in advancing cancer progression, as summarized in Figure 6. HOXA10-AS interacts with miRNAs and acts as a ceRNA to sponge miRNAs. This process inhibits miRNA repression of downstream target genes. Additionally, HOXA10-AS has demonstrated direct interactions with RNA and proteins; hence, HOXA10-AS has an influence on transcriptional and post-transcriptional regulation. Moreover, the downstream regulation of HOXA10-AS primarily occurs through the activation of multiple signaling pathways (Figure 7). HOXA10-AS is involved in cancer progression through the activation of the PIK/AKT, NF-κB, Wnt/β-catenin, Notch, MAPK, and Hippo signaling pathways. These mechanistic investigations provide new targets and methods for therapeutic interventions.

Additional research is still required to fully understand the role of HOXA10-AS in cancer. Although the GEPIA2 and UCSC Xena databases showed that HOXA10-AS was differentially expressed in many different tumor tissues, which implies that HOXA10-AS may be involved in cancer progression, some of data were missing in GEPIA2 databases, such as in LUAD. However, a study has been demonstrated that HOXA10-AS expression was upregulated in LUAD tissues and cells, and the upregulated HOXA10-AS could promote cell proliferation and metastasis (Sheng et al., 2018). Therefore, the results of the database can be used as a comprehensive reference, and more *in vivo* and *in vitro* experiments are needed to validate the potential cancer-promoting or cancer-suppressing effects of HOXA10-AS in cancers. Moreover, lncRNAs were tissue-specific (Della Bella, Koch, and Baerenfaller, 2022), its biological function and regulatory mechanisms in many other common malignancies, such as genitourinary cancers, have not been extensively studied. Furthermore, HOXA10-AS studies in cancers are currently centered around ceRNA regulation, and fewer non-ceRNA explorations have been conducted. Some studies have revealed that lncRNA regulatory mechanisms are very diverse. For example, lncRNA SWINGN promotes tumor progression by binding to SMARCB1 and recruiting chromatin modifiers to the promoter region of GAS6 by forming the SWI/SNF complex (Grossi et al., 2020). LncRNA TUG-1 is involved in R-loop formation regulation at microsatellite repeat regions, thereby control cancer cell proliferation and apoptosis (Suzuki et al., 2023). Additionally, there are problems of low bioavailability and immune-mediated side effects of lncRNA for treatment. And it is necessary to study it in combination with immune molecular mechanisms (Nemeth et al., 2023). Therefore, even though many new therapeutic targets have been discovered, further improvements in drug delivery methods are still required.

In conclusion, lncRNA HOXA10-AS plays a pivotal role in cancer biology and can be utilized as a potential diagnostic and prognostic biomarker for clinical treatment. HOXA10-AS may also serve as a viable therapeutic target since it influences cancer cellular processes. Moreover, further research into the detailed molecular mechanisms of HOXA10-AS in cancer, as well as clinical trials, is still required.
